# New advances and trends in mass spectrometry instrumentation and technology, part I: introduction, single-cell analysis, metabolomics, lipidomics, and multi-omics integration

**DOI:** 10.3389/fmolb.2026.1841624

**Published:** 2026-06-19

**Authors:** Andras Szeitz, Patrick Batoon, Paul R. S. Baker, LeRoy B. Martin

**Affiliations:** 1 Genome Science and Technology Program, University of British Columbia, Vancouver, BC, Canada; 2 Agilent Technologies, Inc., Santa Clara, CA, United States; 3 Sciex, Seattle, WA, United States; 4 Waters Corporation, Milford, MA, United States

**Keywords:** mass spectrometry, round-table discussion, Agilent Technologies, Inc., Sciex, Thermo Fisher Scientific, Waters Corporation

## 1 Introduction

The turn of the 19th and 20th centuries witnessed a series of transformative discoveries that laid the foundation for modern mass spectrometry, including Röntgen’s observation of X-rays in 1895 (Röntgen, 1895), Thomson’s identification of the electron in 1897 (Thomson, 1897), and Wien’s discovery of positive rays, later recognized as protons (Rutherford, 1919). These developments enabled Thomson to construct the first mass spectrometer in 1912 (Thomson, 1912; Thomson, 1913), an instrument subsequently refined by Aston and others (Griffiths, 2008; Münzenberg, 2013). Over the following decades, rapid technological innovation led to the development of time-of-flight analyzers (Wiley and McLaren, 1955), quadrupole and ion-trap instruments (Paul and Steinwedel, 1953; Futrell and Miller, 1966; Yost and Enke, 1978), and Fourier transform ion cyclotron resonance mass spectrometry (Comisarow and Marshall, 1974), among many other advancements that define today’s high-performance platforms. In recent years, the demand for mass spectrometry instrumentation has grown substantially, driven by expanding applications in clinical diagnostics, pharmaceutical development, biotechnology, environmental monitoring, food safety, and multi-omics research (Seger and Salzmann, 2020; Birhanu, 2023). In particular, the increasing adoption of proteomics, metabolomics, lipidomics, and single-cell analysis has created a need for instruments with higher sensitivity, throughput, robustness, and quantitative accuracy (Momenzadeh and Meyer, 2025). At the same time, major progress has been made in the automation of mass spectrometry workflows, including automated sample preparation, integrated liquid chromatography–mass spectrometry platforms, real-time data processing, and standardized analytical protocols that have accelerated mass spectrometric applications in routine clinical laboratories (Vogeser and Habler, 2026). At the same time, instrument miniaturization has also produced more compact mass spectrometers compatible with field applications such as environmental surveillance, forensic investigations, food authentication, and point-of-care testing (Smith et al., 2024; Pond et al., 2026). Combined, these developments are reshaping the role of mass spectrometry from a specialized research technology into a broadly accessible analytical platform with applications spanning laboratories, clinics, and on-site analyses. To explore these developments and the challenges accompanying them, we brought together representatives from major mass spectrometry manufacturers for a round-table discussion focused on emerging technologies, data standardization, automation, accessibility, and future directions for the field. The discussion also addressed the importance of collaboration among academia, industry, clinicians, and regulatory agencies, as well as education and training for the next-generation of scientists. This paper is divided into two parts: Part I (the current paper) covers research applications and technical gaps, including single-cell analysis, metabolomics, lipidomics, and multi-omics integration. And Part II addresses data management and bioinformatics, and collaboration and industry needs. This paper is divided into two parts: Part I (the current paper) covers research applications and technical gaps, including single-cell analysis, metabolomics, lipidomics, and multi-omics integration. And Part II addresses data management and bioinformatics, and collaboration and industry needs. The discussion was published as a webinar, and here we present a summary of that digital event. The discussion was published as a webinar, and here we present a summary of that digital event. The discussion was published as a webinar, and here we present a summary of that digital event.

## 2 Research applications and gaps: single-cell analysis, metabolomics, lipidomics, and multi-omics integration

### 2.1 What are the technical barriers that limit mass spectrometry use in single cell analysis, metabolomics, lipidomics, and volatilomics?

Single-cell omics must be evaluated separately, as metabolomics, lipidomics, and proteomics have different requirements. Single-cell lipidomics is feasible with current technology because lipid concentrations are high enough to translate to single-cell levels. However, sensitivity and sample handling continue to be a problem for single-cell proteomics, where sparse material limits chromatographic separation, etc. Effective isolation, extraction, and measurement of a single cell require techniques with wide linear dynamic ranges -spanning six to seven orders of magnitude -to detect both trace and highly abundant molecules within the same analysis. Single-cell omics begins with defining the biological question and then selecting the technologies capable of answering it. Nowadays, single-cell RNA-seq is routine while single-cell metabolomics, lipidomics, and proteomics are still catching up. Mass spectrometry faces problems that RNA-seq does not have to solve, particularly the lack of amplification. This makes hardware sensitivity critical, rather than sample-prep amplification, because every molecule from a single cell should reach the instrument. Analytical goals also define requirements, where targeted assays can be extremely sensitive, offering absolute quantitation, whereas discovery workflows capture broad analyte coverage. Advances in nano-flow chromatography and highly sensitive ionization interfaces have significantly improved detection, especially when separations and handling are performed as close to the ion source as possible. However, answering biological questions at single-cell resolution brings the challenge of stoichiometry. Are we measuring a single or ten molecules? In bulk samples, averages can be calculated but detecting that same molecule in a single cell is often far more challenging. Subcellular lipidomics pushes this further, as certain lipids are restricted to specific organelles, such as cardiolipin in mitochondria, requiring measurements at subcellular level. Single-cell volatilomics presents additional difficulties as detecting volatile organic compounds produced by a single cell requires high sensitivity, wide range, and adequate sample-handling to ensure analyte detection. As low-flow technologies reduce droplet size and enhance ionization efficiency, ionization chemistry itself becomes the main question. Biomolecules compete for ions or charges, and the challenge is how to ionize everything, and once everything is ionized, how you prevent them from interacting with one another. There are huge technical barriers, and we need to think beyond typical electrospray and look into more selective secondary ionization as a downstream separation beyond chromatography. For metabolomics samples, the main agenda has been detecting thousands of features for years even though only a few were actually identified. It raises the question with what level of specificity we want to measure our compounds? The data collected with mass spectrometry needs to be translated to meaningful knowledge. In single-cell metabolomics, lipidomics, or proteomics, one question to ask can be whether we are truly identifying molecules, or just collecting features? Any technology can generate single-cell signals, but the key challenge is turning those signals into relevant identification and quantitation. To answer the questions whether a signal is real, noise, or an adduct, we need MS/MS and product-ion analysis, which impacts sensitivity and many other factors. The level of specificity needed in single-cell work is a question for biologists. The ion statistics of a mass spectrometer collecting sufficient ions for accurate, precise quantification also raises key questions as to how many single cells are needed, and how much biological variability exists between identical cells in different cell-cycle states. Because these factors are tightly regulated, studies often require scaling from one sample to potentially millions of cells, as seen in other single-cell technologies that demand high throughput for robust statistical power.

Polarity switching is routinely used in lipidomics, alternating between negative and positive ionization modes during acquisition. Running each mode separately may give us more detailed data, however, we can analyze a single cell only once, otherwise we have to split it in two with one experiment for positive, one experiment for negative ionization, which would mean that we are analyzing half a cell in each mode. Fast polarity switching on modern mass spectrometry instruments helps overcome this by allowing both modes to be captured in a single shot, which significantly improves single-cell lipidomics performance. In bulk samples, where material is abundant, separate runs in positive and negative mode, and improving peak detection by using reverse phase, and HILIC, ion chromatography, gas chromatography are popular, but when we have limited sample amounts, it may not be possible or would decrease performance if we only get one shot. Polarity switching can also help maintain instrument performance. Extended analysis in a single ionization mode can lead to gradual accumulation of analyte deposits along the ion optics rail, creating a layer that can impede ion transmission into downstream ion optics. Switching polarity helps discharge this buildup and restores sensitivity. Whether we are using fast polarity switching or include it in the method as a washing out step, it really helps the system with fundamental reasons behind it.

If a single cell really provides only one analytical shot, integrating multiple omics becomes challenging. The emphasis may be on metabolomics and lipidomics, but proteomics is also important. The broader goal is to extract metabolomic, lipidomic, and proteomic information from the same cell, yet a practical question remains why not using more cells instead of only one cell. What if one cell lacks the molecule of interest, but maybe ten cells have it? At what point does single-cell analysis reach diminishing returns? Maybe, there is an endpoint to lipidomics, metabolomics, proteomics, we just have not figured it yet. Single-cell imaging adds another dimension, capturing chemical information directly *in situ* while confirming spatial origin with an optical image. Cells constantly change molecular composition, but avoiding physical separation can simplify interpretation. Ultimately, true single-cell analysis may not be the end point, and for many questions, subcellular or spatially resolved analysis offers greater biological information. Techniques, such as deep visual proteomics, where we work with smaller subsections of a tissue and analyze them in combination with microscopy overlaying multiple pieces of information, such as staining with proteomics or staining with some omics measurement on mass spectrometry. And this is an extremely promising field that will advance our understanding of biology.

### 2.2 How do you see the integration of mass spectrometry with other omics platforms evolving in the next few years?

In some instances, genomics, pathology, proteomics, and small-molecule analysis overlap with multi-omics. The challenge is how to bring these disciplines together into a single data stream capable of describing individuals or populations. Multi-omics tracks the central dogma of biology how genetics affects disease, proteins, small molecule expression, etc. The biggest challenge is merging the data from different fields of science who speak different languages essentially and often have no expertise amongst each other. As analytical chemists, we may understand molecules and biology, but many of us may lack training in bioinformatics or genomic data interpretation, and this gap complicates integration. The more we learn about multi-omics, the clearer it becomes how complex the biological question is. DNA turns into mRNA, which is translated into proteins, which get modified post translationally, degraded and regulated in spatial contexts. Metabolites are generated through networks of pathways rather than simple one-enzyme–one-product synthesis. As we move from genomics to transcriptomics, proteomics, and metabolomics, we can witness a massive expansion of information, and without shared identifiers and standardized ways of describing these data, integration becomes nearly impossible. Maintaining the solid motivation to connect these domains, many companies now develop workflows spanning multiple omics layers. Emerging AI and deep-learning approaches offer promise for making sense of these massive, complex datasets, enabling new insights through advanced visualization and pattern recognition. AI may certainly help in unbiased and unsupervised data analysis. For example, in untargeted analysis, PCA plots are unsupervised, however, some people also prefer a PLS-DA, which is supervised. There are various advantages and disadvantages of those two approaches, and one of the promises of AI is that it can create new models or new understandings of what dependent and independent variables might be by cross correlations across these seemingly unrelated data, which certainly opens new opportunities. AI could be a powerful tool for normalizing data sets so they can be correlated. Proteomics measures milligrams of protein, lipidomics uses millilitres of plasma or another matrix and extract the compound. AI will probably help deal with the massive data sets approaching terabytes in a single sample, but there should be a strategy to assist with fundamental issues, such as normalizing the data sets, so that they can be correlated, then having a unified way of comparing proteins to lipids to which AI may offer some exciting solutions. One point that emerged in the discussion is that transcriptomics and proteomics data sometimes do not correlate perfectly. The familiar model of one gene producing one mRNA that produces one protein may be simplistic when evaluated from the multi-omics perspective. In practice, even when a protein is known to catalyze the formation of specific lipid metabolites, the expected downstream lipid changes may not appear, which may be because numerous additional genes and proteins interplay in the pathways. In such networks, AI could assist by identifying the relevant pathway among the possibilities. Hence, integrating AI tools into mass spectrometry workflows could be a useful development.

An example of multilateral biochemical pathways could be the studies in eicosanoids, where phospholipids release arachidonic acid, which cyclooxygenase converts into prostaglandins, a mechanism that underlies the action of NSAIDs, such as Advil and Tylenol. However, in certain tissues, an alternative route exists where arachidonic acid is first converted into 2-arachidonoylglycerol and later hydrolysed to eicosanoids. This showed that biochemical pathways may interconnect through various biochemical reactions, and their activity may be state and tissue dependent. With advanced supervised or unsupervised learning applied to large datasets, AI’s function will likely be a scientific copilot, supporting, not replacing, the researcher. AI still lacks the conceptual understanding of biochemical context and dependency, so human guidance remains essential. But as an augmenting tool, especially in pathway mapping, it could be transformative.

The analytical approach we choose fundamentally shapes our outcomes. In small-molecule analysis, early biochemical pathways were elucidated without mass spectrometers (relying mainly on chemists), but today, targeted analyses using triple-quadrupole mass spectrometers are foundational. Although broad, discovery-style experiments are powerful, hypothesis-driven targeted measurements are often the most effective way to answer specific biological questions. After years of trials, we may have realized that asking the question what was in the sample may not be the correct query. Instead, starting with a targeted sequence, while asking the questions whether compound A, B, or C is present may ultimately lead to the correct answer. Lipidomics illustrates this shift well. Early shotgun approaches were wide and semi-targeted, but the field has increasingly moved toward targeted approaches because current software still struggles with isomers, isobars, and complex data processing. Proteomics is further ahead, but metabolomics and lipidomics remain challenging as we continue refining how to measure these molecules accurately.
